# Abdominal Muscle Wall Abscess as a Rare Extra-abdominal Complication of Acute Diverticulitis: A Case Report and Review of Literature

**DOI:** 10.7759/cureus.87530

**Published:** 2025-07-08

**Authors:** Hannah N Ortega Aranda, Marco Antonio Urbina Velázquez, Pablo Orozco Obregón, José Emiliano González Flores

**Affiliations:** 1 Department of Surgery, American British Cowdray (ABC) Medical Center, Mexico City, MEX; 2 School of Medicine and Health Sciences, Tecnológico de Monterrey (ITESM), Mexico City, MEX

**Keywords:** abdominal muscle wall abscess, ct imaging, diverticulitis, extra-abdominal complication, percutaneous drainage

## Abstract

Diverticulitis may lead to intra-abdominal abscesses; however, extra-abdominal complications such as abdominal muscle wall abscesses are exceedingly rare. These abscesses can present weeks after conservative treatment, often with nonspecific symptoms that delay diagnosis. Understanding rare extension pathways of diverticular inflammation is essential for early recognition and management. We report a case of a 48-year-old male with grade III obesity who presented with a painful abdominal mass one month after an episode of sigmoid diverticulitis. Contrast-enhanced computed tomography revealed a loculated abscess in the anterior abdominal muscle wall. The patient was successfully managed with ultrasound-guided percutaneous drainage and targeted antibiotic therapy, achieving full recovery without surgical intervention. This case highlights the importance of early imaging and individualized, minimally invasive treatment in select patients. Clinicians should maintain a high index of suspicion for atypical presentations in unresolved diverticulitis.

## Introduction

Diverticular disease is a common gastrointestinal condition with global prevalence ranging from 20% to 60% [1]. Among affected individuals, approximately 4% develop acute diverticulitis, and 15-20% of these cases may progress to complications such as abscess formation [2]. While most diverticular abscesses remain confined to the peritoneal cavity, rare extra-abdominal presentations have been documented, including abscesses in the thigh, hip, gluteal region, and abdominal muscle wall [1].

Extraperitoneal abscesses typically present weeks after an episode of perforated diverticulitis. Their clinical manifestations are often nonspecific, characterized by localized abdominal pain and a small palpable mass (<5 cm), which may delay diagnosis [3].

The exact pathogenesis of these extra-abdominal abscesses is not fully understood. Proposed mechanisms include the spread of inflammation through neurovascular bundles, inguinal canals, muscular planes, and fascial layers, allowing communication between the peritoneal cavity and extraperitoneal sites [1].

In the diagnostic approach, ultrasonography may serve as an initial tool, potentially identifying increased echogenicity, discontinuity of fascial planes, or subcutaneous air. However, contrast-enhanced computed tomography (CT) is considered the gold standard due to its high sensitivity in delineating abscess extension and identifying potential intra-abdominal communications [4].

Abscess size is a key factor in therapeutic decisions. Collections smaller than 3 cm may resolve with antibiotics alone, whereas larger abscesses usually require percutaneous drainage (PCD) in addition to antibiotic therapy to achieve clinical resolution [3,4].

The objective of this report is to describe an abdominal muscle wall abscess as an extremely rare extra-abdominal complication following an episode of acute diverticulitis.

## Case presentation

A 48-year-old male with significant medical comorbidities, including grade III obesity and limited mobility requiring a walker, leading to a predominantly sedentary lifestyle, presented with a prior episode of acute diverticulitis in June 2024. This episode was complicated by a 25 cc perisigmoid abscess and managed conservatively at an external facility with oral ciprofloxacin and metronidazole for seven days, resulting in symptom remission. However, the patient presented again in July 2024 with a recurrence, reporting severe colicky pain in the left flank and iliac fossa, accompanied by nausea and four episodes of gastrointestinal emesis. On arrival, he was somnolent, disoriented to time and place, exhibited generalized edema, reduced muscle strength, and was unable to ambulate. Physical examination revealed a painful, palpable mass measuring approximately 11 cm in the left iliac fossa.

**Figure 1:**
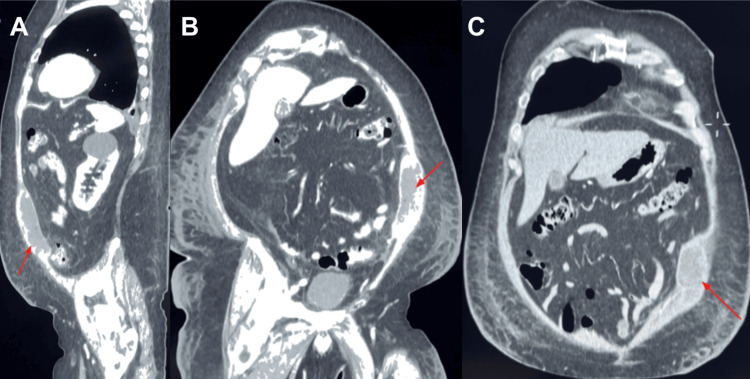
Contrast-enhanced CT images showing abdominal wall abscess. (A) Sagittal view reveals a loculated hypodense collection involving the left anterior abdominal wall (red arrow). (B-C) Axial sections demonstrate the abscess extending through the muscular layers (rectus and obliques), abutting adjacent bowel structures. The collection appears well-demarcated and heterogeneous, consistent with a mature abscess cavity (red arrows).

Initial laboratory tests showed mildly increased inflammatory markers, including CRP and procalcitonin, with white blood cell and neutrophil counts within normal limits. Details are summarized in Table 1. A contrast-enhanced abdominopelvic CT scan revealed multiple colonic diverticula, predominantly in the sigmoid segment, and a large loculated collection within the left anterior abdominal muscle wall (Figure 1). The collection involved the left rectus and oblique muscles, extended from the flank to the iliac fossa, measured 11.4 × 6.6 cm, and had intimate contact with the adjacent small bowel and descending colon.

**Table 1:** Initial and follow-up laboratory values.

Laboratory parameter	Normal range	Initial value	Follow-up value
White blood cell count (WBC)	4.0-10.0 ×10^9^/L	6.02 ×10^9^/L	5.8 ×10^9^/L
Neutrophil count	2.0-7.5 ×10^9^/L	4.29 ×10^9^/L	4.4 ×10^9^/L
C-reactive protein (CRP)	<5 mg/L	37 mg/L	32 mg/L
Procalcitonin	<0.1 ng/mL	0.15 ng/mL	-

**Figure 2:**
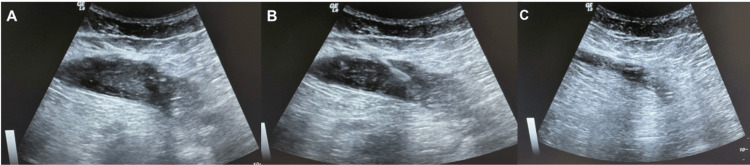
Ultrasound evaluation of the abdominal wall abscess. (A-B) Transverse greyscale images demonstrate a heterogeneous, hypoechoic, loculated collection within the left lower abdominal wall, suggestive of an organized abscess. (C) Post-drainage view showing the catheter tract and partial resolution of the collection with decreased fluid content.

Empirical intravenous meropenem (1 g every eight hours) was initiated. Ultrasound-guided PCD (Figure 2) was performed using a 12 French (Fr) Dawson-Mueller catheter, yielding 58 mL of purulent fluid that cultured positive for Gram-positive cocci.

A follow-up CT scan on post-drainage day 2 confirmed near-complete resolution of the collection without evidence of fistulous communication with the abdominal cavity. The patient demonstrated steady clinical improvement with decreasing serohematic drainage output, which remained below 19 mL daily. On post-drainage day 4, drainage was minimal (5 mL, serous), and the patient was discharged with a 10-day course of intramuscular ertapenem (1 g/day).

By post-drainage day 19, the patient remained clinically stable, with inflammatory markers slightly elevated and leukocyte counts within normal limits. Given the minimal drainage, the catheter was removed. The patient was advised to undergo a colonoscopy and continue with outpatient follow-up, including clinical evaluation and a scheduled follow-up CT scan.

## Discussion

Abdominal muscle wall abscesses represent an exceedingly rare extra-abdominal complication of acute diverticulitis. This case demonstrates an atypical evolution from a suboptimally treated, conservatively managed episode of sigmoid diverticulitis to the formation of a delayed, loculated abscess within the anterior abdominal muscle wall. This case illustrates the diagnostic complexity and therapeutic challenges of an atypical complication of sigmoid diverticulitis. A successful outcome was achieved through image-guided PCD and targeted antimicrobial therapy, avoiding the need for surgical resection. While intra-abdominal abscess formation is well recognized, extra-abdominal extension to the abdominal wall remains exceptionally uncommon. The first report of such a phenomenon was published in 1926 by Rodlaha, describing a subcutaneous and subdiaphragmatic abscess following a perforated gastric ulcer [1].

Rafailidis et al. reported a case of a 73-year-old man presenting with pain and a visible mass in the lower left quadrant, where diagnostic imaging, ultrasound, and CT revealed a bilocular abscess originating from ruptured sigmoid diverticulitis and extending into the abdominal muscle wall. The patient responded well to local drainage and antibiotics [2]. Similarly, Sakurai et al. described jejunal diverticulitis leading to an abdominal muscle wall abscess, necessitating surgical resection [3]. Alvarez et al. also documented abdominal wall abscesses due to jejunal diverticulitis, especially in patients with prior abdominal surgeries [4]. A summary of similar published cases of abdominal wall abscesses secondary to diverticulitis is presented in Table 2, highlighting the variability in underlying bowel segment, abscess location, treatment approach, and outcomes.

**Table 2:** Reported cases of abdominal wall abscesses secondary to diverticulitis.

Author	Year	Type of diverticulitis	Abscess location	Treatment	Outcome
Rafailidis et al. [2]	2013	Sigmoid	Left abdominal wall	Drainage + antibiotics	Full recovery
Sakurai et al. [3]	2005	Jejunal	Abdominal wall	Surgical resection	Good outcome
Alvarez et al. [4]	1995	Jejunal	Abdominal wall	Surgery (drainage/resection)	Resolution

A comprehensive umbrella review by Cirocchi et al. synthesized 12 international guidelines on the management of intra-abdominal diverticular abscesses. Most recommend conservative treatment with bowel rest and intravenous antibiotics for small (<3 cm) collections, and image-guided PCD for larger (3-5 cm) abscesses. Emergency surgery is reserved for patients with generalized peritonitis or failure of non-operative management [5].

A clinical guideline review by You et al. supports this therapeutic stratification, noting that appropriate surgical intervention in cases of generalized peritonitis includes the Hartmann procedure or primary anastomosis with or without diverting ileostomy [6]. Importantly, Gregersen et al. demonstrated that acute surgery in these patients is associated with significantly higher mortality (12.1%) compared to conservative management (1.1%) [7].

Our case aligns with these recommendations. The patient presented with a large, loculated abdominal muscle wall abscess following prior sigmoid diverticulitis, without signs of systemic sepsis or peritonitis. He was successfully treated with ultrasound-guided PCD and targeted antibiotic therapy, thereby avoiding surgical intervention.

Understanding the anatomic pathways through which intra-abdominal infections extend to extra-abdominal compartments, via neurovascular bundles, fascial planes, or postoperative weakness, is crucial for timely diagnosis and proper management of such rare entities [2].

This case underscores the importance of maintaining a high index of suspicion for atypical presentations of diverticular disease, particularly in patients with persistent or recurrent symptoms following initial conservative treatment. While diverticulitis is a frequent cause of intra-abdominal abscesses, rare cases of infection spreading to extra-abdominal compartments such as the anterior abdominal muscle wall have been documented, even in the absence of overt perforation or fistula formation [2-4].

Diagnostic imaging plays a pivotal role in such scenarios. Although ultrasonography can serve as an initial tool, contrast-enhanced CT remains the gold standard for assessing the extent of disease, identifying abscess formation, and ruling out complications such as fistulous tracts or generalized peritonitis [5,8]. Early use of CT in patients with unexplained or evolving abdominal symptoms post-diverticulitis may facilitate timely diagnosis and intervention, thus minimizing morbidity.

Our patient’s successful management with PCD and antibiotic therapy aligns with international guidelines for the management of diverticular abscesses, which recommend non-operative treatment for stable patients with abscesses larger than 3 cm and no signs of peritonitis [6,7]. This reinforces the importance of stratifying treatment based on abscess size, location, and patient stability.

Furthermore, it emphasizes the role of structured clinical follow-up in cases of complicated diverticulitis, as delayed complications may arise after apparent initial resolution. A multidisciplinary approach involving surgery, radiology, and infectious disease specialists can optimize outcomes and prevent unnecessary surgical interventions in stable patients [7,9].

The current gold standard for evaluating suspected complications of diverticulitis, such as abscess formation or fistulization, is contrast-enhanced CT. CT not only provides accurate anatomic localization and characterization of abscesses but also detects extraperitoneal spread and guides interventional planning. It outperforms ultrasonography in sensitivity and specificity, particularly in obese or complex patients [8,10].

When it comes to treatment, international guidelines recommend stratification based on the size of the abscess and the presence of systemic signs. For abscesses <3 cm, conservative management with bowel rest and intravenous antibiotics is typically sufficient. Abscesses between 3 and 5 cm generally require image-guided PCD in addition to antibiotics, whereas surgical intervention is indicated in the presence of generalized peritonitis, clinical instability, or failure of non-operative treatment [5,6].

In our case, the patient met the criteria for non-operative management; he was hemodynamically stable, had no peritonitis, and imaging confirmed a localized collection without a fistula. Image-guided drainage using a 12 Fr catheter was successful, consistent with established protocols and evidence favoring percutaneous intervention for mature, well-defined collections [7]. This management strategy aligns with the contemporary shift toward minimally invasive approaches in the treatment of diverticular abscesses.

Although our patient was successfully treated without surgery, it is important to acknowledge surgical options for patients presenting with different clinical profiles. In cases where surgical intervention is necessary, options include Hartmann’s procedure or primary anastomosis with or without protective ileostomy, depending on intraoperative findings and patient stability. Several studies have suggested that the use of laparoscopic peritoneal lavage may also be an alternative in carefully selected patients with purulent peritonitis, although this remains controversial [11,12].

This case highlights the importance of timely imaging, risk stratification, minimally invasive intervention, and targeted antibiotic therapy, leading to a favorable outcome without the morbidity of surgery.

The rarity of abdominal wall abscesses secondary to acute diverticulitis raises important questions regarding their pathogenesis and risk factors. One plausible hypothesis is that local inflammatory processes from the sigmoid colon may extend beyond the peritoneum through anatomical planes such as neurovascular bundles, fascial planes, or pre-existing tissue weaknesses, even in the absence of overt perforation or identifiable fistulous tracts [2-4].

An alternative hypothesis is that delayed or incomplete resolution of a pericolic abscess managed conservatively may allow the infection to dissect through soft tissues over time, leading to extra-peritoneal tissue involvement. This may be especially relevant in patients with predisposing conditions such as obesity, limited mobility, or prior abdominal wall compromise due to surgery, trauma, or inflammation [5,7].

This case report presents several notable strengths. First, it documents an exceptionally rare complication of acute diverticulitis, an abdominal muscle wall abscess, which adds valuable insight to the limited literature on extra-abdominal manifestations of this disease. Second, the inclusion of both ultrasonographic and contrast-enhanced CT imaging provides objective and sequential diagnostic evidence, supporting the diagnosis and follow-up. Third, the successful management with PCD and targeted antibiotic therapy highlights the applicability and efficacy of conservative treatment in select patients, consistent with current guidelines for abscesses greater than 3 cm. Finally, the case aligns well with contemporary recommendations for non-operative management, reinforcing the clinical utility of established treatment algorithms even in atypical presentations.

This case underscores the need for clinical vigilance in patients with diverticulitis who fail to respond fully to conservative treatment, as atypical presentations, though rare, can lead to delayed diagnosis and increased morbidity if not promptly identified.

From a diagnostic perspective, the case underscores the central role of contrast-enhanced CT as the gold standard in identifying not only intra-abdominal complications but also their potential extension beyond the peritoneal cavity. While ultrasonography may assist in guiding interventions such as PCD, CT remains critical for surgical planning and for evaluating anatomical relationships.

In terms of management, the case validates current guidelines advocating for non-operative treatment of stable patients with well-defined abscesses larger than 3 cm. The successful outcome achieved with PCD and antibiotic therapy supports the growing body of evidence favoring less invasive approaches when clinically appropriate. Moreover, the absence of surgical morbidity in this patient, who had multiple comorbidities and reduced functional status, highlights the relevance of tailoring interventions based on individual risk profiles.

Ultimately, this case highlights the necessity of individualized follow-up strategies in patients managed conservatively for diverticulitis, particularly those with complex risk profiles. It reinforces the importance of clinical vigilance in detecting delayed or atypical complications that may otherwise be missed.

Future research should aim to systematically document and analyze similar rare cases to better understand the anatomical and pathological mechanisms driving extra-abdominal abscess formation. Multicenter registries, coupled with advanced imaging studies, may help identify predictive factors for atypical presentations and treatment failure. Prospective studies comparing conservative and surgical approaches in rare abscess locations could further refine treatment algorithms and improve patient outcomes.

## Conclusions

Abdominal muscle wall abscesses are an exceptionally rare extra-abdominal complication of acute diverticulitis, posing a significant diagnostic and therapeutic challenge. This case illustrates an uncommon clinical progression from a conservatively managed sigmoid diverticulitis to delayed abscess formation within the anterior abdominal wall, in the absence of peritonitis or overt fistulization. Timely use of contrast-enhanced CT imaging was essential for accurate diagnosis and procedural planning, while successful image-guided PCD and targeted antibiotic therapy obviated the need for surgical intervention. The case reinforces the importance of individualized, minimally invasive management strategies in stable patients, particularly those with comorbidities that increase surgical risk.

Clinicians should maintain a high index of suspicion for atypical abscess locations in patients with unresolved or recurrent symptoms following diverticulitis. Structured follow-up and early imaging are critical to identify delayed complications. This report also underscores the need for multicenter data collection and systematic analysis of rare presentations to better understand their pathophysiology and optimize therapeutic decision-making.
